# 2209. RSV-associated Hospitalizations in Adults Aged ≥18 Years and the Impact of the COVID-19 Pandemic in the United States, October 2018 – February 2022

**DOI:** 10.1093/ofid/ofac492.1828

**Published:** 2022-12-15

**Authors:** Fiona P Havers, Michael Whitaker, Huong Pham, Onika Anglin, Jennifer Milucky, Kadam Patel, Pam Daily Kirley, Elizabeth Austin, James Meek, Evan J Anderson, Maya Monroe, Chloe Brown, Erica Bye, Francesca Pacheco, Grant Barney, Virginia Cafferky, Melissa Sutton, Keipp Talbot, Ryan Chatelain, Susan I Gerber, Gayle Langley, Lindsay Kim, Christopher Taylor

**Affiliations:** CDC, Atlanta, Georgia; CDC, Atlanta, Georgia; Centers for Disease Control and Prevention, Atlanta, Georgia; CDC, GDIT, Atlanta, Georgia; Centers for Disease Control and Prevention, Atlanta, Georgia; Centers for Disease Control and Prevention, Atlanta, Georgia; California Emerging Infections Program, Oakland, California; Colorado Department of Public Health and Environment - Communicable Disease Branch, Denver, Colorado; Connecticut Emerging Infections Program, New Haven, Connecticut; Emory University School of Medicine, Atlanta, Georgia; Maryland Department of Health, Baltimore, Maryland; Michigan Department of Health and Human Services, Lansing, Michigan; Minnesota Department of Health, Saint Paul, Minnesota; University of New Mexico Health Sciences Center, Albuquerque, New Mexico; New York State Department of Health, Albany, New York; University of Rochester School of Medicine and Dentistry, Rochester, New York; Oregon Health Authority, Portland, Oregon; Vanderbilt University Medical Center, Nashville, Tennessee; Salt Lake County Health Department, Salt Lake City, Utah; US CDC, Atlanta, Georgia; Centers for Disease Control and Prevention, Atlanta, Georgia; CDC, Atlanta, Georgia; CDC, Atlanta, Georgia

## Abstract

**Background:**

Respiratory syncytial virus (RSV) is a significant cause of hospitalizations in older adults and typically circulates during the fall and winter in the United States. The COVID-19 pandemic and implementation of nonpharmaceutical interventions (NPIs) including masking, improved handwashing, and social distancing likely impacted RSV circulation. To explore the pandemic’s impact on RSV seasonality and hospitalizations in adults aged ≥18 years, we analyzed laboratory-confirmed RSV-associated hospitalizations through the RSV Hospitalization Surveillance Network (RSV-NET) across four seasons.

**Methods:**

RSV-NET is a population-based surveillance system that collects data on RSV-associated hospitalizations across 75 counties in 12 states. An RSV-NET case is a resident of a defined catchment area who tests positive for RSV through a clinician-ordered test within 14 days prior to or during hospitalization. Surveillance was conducted October–April for the 2018-19 and 2019-20 pre-pandemic seasons and October 2020–September 2021 (2020-21 season). Available data October 2021-February 2022 (ongoing 2021-22 season) are presented.

**Results:**

2,536, 3,195, 618, and 1,758 laboratory-confirmed hospitalizations were identified in adults ≥18 years in 2018-19, 2019-20, 2020-21, and 2021-22, respectively; case counts were 4.1 and 5.2 times higher in 2018-19 and 2019-20, respectively, than in 2020-2021. Hospitalizations peaked in January for pre-pandemic and 2021-22 seasons and in September for 2020-21 (Figure). For all years combined, 16.2%, 23.4%, 33.3%, and 27.1% of all RSV-associated hospitalizations were among those aged 18-49, 50-64, 65-79 and ≥80 years, respectively.

**Figure:**
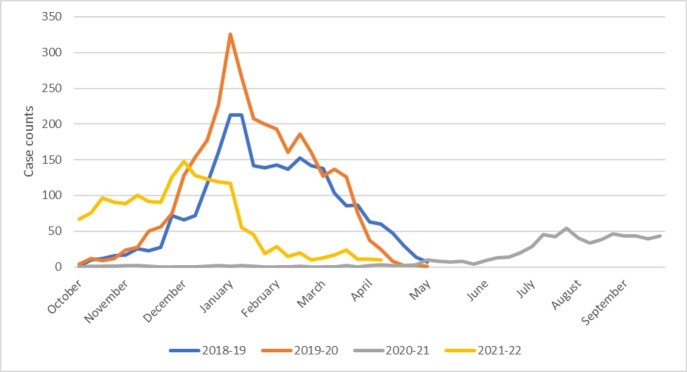

Laboratory-confirmed RSV-associated hospitalizations in adults ≥18 years, October 2018 – February 2022

**Conclusion:**

Laboratory-confirmed RSV-associated hospitalizations in adults were lower during the 2020-21 and 2021-22 seasons compared with pre-pandemic seasons, with a marked change in seasonal patterns in 2020-21, likely because of NPIs implemented during the pandemic. Continued monitoring of RSV-associated hospitalizations will be critical to understand ongoing changes in RSV circulation that resulted from the COVID-19 pandemic and associated NPIs.

**Disclosures:**

**Evan J. Anderson, MD**, GSK: Advisor/Consultant|GSK: Grant/Research Support|Janssen: Advisor/Consultant|Janssen: Grant/Research Support|Kentucky Bioprocessing, Inc: Data Safety Monitoring Board|MedImmune: Grant/Research Support|Medscape: Advisor/Consultant|Merck: Grant/Research Support|Micron: Grant/Research Support|NIH: Funding from NIH to conduct clinical trials of Moderna and Janssen COVID-19 vaccines|PaxVax: Grant/Research Support|Pfizer: Advisor/Consultant|Pfizer: Grant/Research Support|Regeneron: Grant/Research Support|Sanofi Pasteur: Advisor/Consultant|Sanofi Pasteur: Grant/Research Support|Sanofi Pasteur: Data Adjudication and Data Safety Monitoring Boards|WCG and ACI Clinical: Data Adjudication Board **Maya Monroe, MPH**, CDC -Emerging Infections Program: Grant/Research Support.

